# Molecular Pathways Involved in LRRK2-Linked Parkinson’s Disease: A Systematic Review

**DOI:** 10.3390/ijms231911744

**Published:** 2022-10-03

**Authors:** Ailyn Irvita Ravinther, Hemaniswarri Dewi Dewadas, Shi Ruo Tong, Chai Nien Foo, Yu-En Lin, Cheng-Ting Chien, Yang Mooi Lim

**Affiliations:** 1 Centre for Cancer Research, M. Kandiah Faculty of Medicine and Health Sciences, Universiti Tunku Abdul Rahman, Kajang 43000, Selangor, Malaysia; 2 Institute of Molecular Biology, Academia Sinica, Taipei 115, Taiwan; 3 Centre for Biomedical and Nutrition Research, Faculty of Science, Universiti Tunku Abdul Rahman, Kampar 31900, Perak, Malaysia; 4 Department of Population Medicine, M. Kandiah Faculty of Medicine and Health Sciences, Universiti Tunku Abdul Rahman, Kajang 43000, Selangor, Malaysia; 5 Department of Pre-Clinical Sciences, M. Kandiah Faculty of Medicine and Health Sciences, Universiti Tunku Abdul Rahman, Kajang 43000, Selangor, Malaysia

**Keywords:** Parkinson’s disease, mechanism, LRRK2

## Abstract

Parkinson’s disease is one of the most common neurodegenerative diseases affecting the ageing population, with a prevalence that has doubled over the last 30 years. As the mechanism of the disease is not fully elucidated, the current treatments are unable to effectively prevent neurodegeneration. Studies have found that mutations in Leucine-rich-repeat-kinase 2 (LRRK2) are the most common cause of familial Parkinson’s disease (PD). Moreover, aberrant (higher) LRRK2 kinase activity has an influence in idiopathic PD as well. Hence, the aim of this review is to categorize and synthesize current information related to LRRK2-linked PD and present the factors associated with LRRK2 that can be targeted therapeutically. A systematic review was conducted using the databases PubMed, Medline, SCOPUS, SAGE, and Cochrane (January 2016 to July 2021). Search terms included “Parkinson’s disease”, “mechanism”, “LRRK2”, and synonyms in various combinations. The search yielded a total of 988 abstracts for initial review, 80 of which met the inclusion criteria. Here, we emphasize molecular mechanisms revealed in recent in vivo and in vitro studies. By consolidating the recent updates in the field of LRRK2-linked PD, researchers can further evaluate targets for therapeutic application.

## 1. Introduction

Parkinson’s disease (PD) is the second most common neuropathology after Alzheimer’s disease [1]. It has the fastest-growing prevalence and most deaths among neurological disorders, affecting nearly 6.1 million ageing people in 2016 globally, which is more than doubled from the past generation [2]. PD is a multisystem disease that affects the nervous system, especially the central nervous system (CNS), with the pathological characteristic of losing more than 50% of the dopaminergic neurons in the substantia nigra pars compacta [3,4]. Dopaminergic neurons are the main source of producing rich dopamine (DA). The depletion of DA manifests in the progressive degeneration of motor mobility with the clinical symptoms of bradykinesia, rigidity, rest tremor, and postural instability [1,5]. In addition, the neuropathology of PD also exhibits heterogeneous clinical symptoms in non-motor function (deficit in olfactory, depression and cognition, rapid eye movement sleep behaviour disorder, constipation, and central autonomic control) at all stages of the PD pathological process, even predating the motor symptoms [6].

PD is considered as multifactorial neuropathology with complex aetiology, which was found to be highly correlated with ageing, abnormal alpha-synuclein (α-Syn) accumulation in dopaminergic neurons, exposure to polluted environment, and genetic susceptibility [1,3,7]. However, the risk of PD pathogenesis was found to interplay with multiple different factors and eventually give rise to different clinical symptoms. More often, the disease mechanisms may not be completely identical in all PD patients. Hence, the complexity of PD disease increases the difficulty to reveal a clearer pathogenesis pathway.

To date, there are only approximately 10–15% of patients reported as family history of PD symptoms, while the remaining 85–90% of the PD population are classified as sporadic PD [1,8]. More than 20 genes were found to be involved in familial PD, in which the penetrant mutation is rare, with an occurrence of 2% in the PD population [7,8,9]. These mutations present a broad range of risks and demonstrate heterogeneous mechanisms associated with PD. Among the PD causative genes, the 51-exon Leucine-rich repeat kinase (LRRK2) gene is the most common mutation, where the variants are reported in 0.7% of all the people showing PD symptoms [8]. Hence, a missense mutation in LRRK2 G2019S received the most attention because it appears to be the most penetrant mutation in the autosomal dominant PD and sporadic PD, with an influence of 40% and 10%, respectively [4,5,10]. Moreover, the occurrence of LRRK2 variants affecting a wide span of diverse ethnicities in different geographic distributions further define its role of genetic susceptibility in the PD population [11].

**Figure 1:**
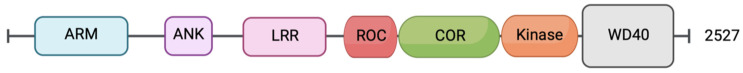
Simple schematic of LRRK2 protein structure (2527 amino acids long) and its domains. ROC, COR, and kinase make up the enzymatic domains of LRRK2, whereas ARM, ANK, LRR, and WD40 control protein–protein interactions. Created with BioRender.com (accessed on 25 July 2022).

The protein encoded from the LRRK2 gene belongs to the Roco protein family with the presence of the Ras of Complex (ROC) G-domain adjacent to the C-terminal of the ROC (COR) linker. Other than the ROC-COR domain, LRRK2 also consists of N-terminal ankyrin-like repeats (ANK), armadillo repeats (ARM), Leucine-rich repeats (LRR), and kinase and C-terminal WD40 repeats (Figure 1) [10,12,13]. Therefore, this multi-domain LRRK2 protein manifests multifunctional activities through exhibiting catalytic activities as GTPase and kinase, as well as interacting with diverse proteins. 

There are nearly 100 mutations identified at the different domains of LRRK2, in which some are related to the pathogenesis of PD, including G2019S, R1441C/G/H, I2020T, and Y1699C. The mutation sites are highly conserved and segregate in families with PD disease [5,8,10]. These mutations lead to gain-of-function mechanisms, where kinase activity of LRRK2 will increase to phosphorylate a group of Rab proteins in different subcellular localities, thus affecting downstream pathways to drive PD pathogenesis [14]. Interestingly, in in vitro and in vivo studies, knocking out LRRK2 recapitulated aspects of PD pathogenesis such as dopaminergic neuron loss, accumulation of α-Syn, impairment of protein degradation, and dysregulation of autophagy [15,16,17,18]. However, there is a lack of evidence of phenotypic impact, as there was no increase in the risk of PD in humans carrying loss of function variants of LRRK2 [19].

Many studies also show that the aberrant function of LRRK2 to PD is not necessary by mutation alone, instead, some upstream factors such as Rab29 and α-Syn could exacerbate the activity of LRRK2 [14,20,21]. Therefore, evidence suggests that the mutant LRRK2 is inter-related with other proteins and impacts diverse cellular biological processes, increasing the complexity and enigma of the understanding of PD pathogenesis.

As the world population ages and life expectancy increases, it is crucial to explore and address the yet unresolved health challenge of PD to prevent and improve PD prognosis. Understanding the interaction and mechanism involved in PD is key to defining the pathophysiology of the disease. Since LRRK2 is a pleiotropic factor in PD at both genetic and molecular levels, this review aims to understand the pathogenesis behind LRRK2-linked PD through its molecular mechanism associated with upstream and downstream effects on multiple biological processes. The in vivo and in vitro studies involving the mechanism of PD were systematically reviewed to provide insight for a better therapeutic option in the future.

This paper systematically searched for targets and specific mechanisms of PD to consolidate novel and established targets of LRRK2.

## 2. Methods

This review was conducted following the Preferred Reporting Items for Systematic Reviews and Meta-Analyses (PRISMA) statement [22].

### 2.1. Search Strategy

A systematic literature search was performed using the databases: PubMed, Medline, SCOPUS, SAGE, and Cochrane. Only publications within January 2016 to July 2021 were included in this systematic review.

The search strategy comprised combinations of the keywords: “Mechanism” AND “LRRK2” AND “Parkinson’s disease” including the synonyms of each keyword, as shown in Table 1. These terms were searched for in the titles, abstracts, and full texts where applicable. Articles that were not published in English were not included.

### 2.2. Study Selection

Two authors independently extracted and proofread the titles and abstracts of each article. The inclusion criteria comprised of human in vivo and in vitro studies related to mechanisms of pathogenesis for LRRK2-linked PD and studies related to mechanisms of PD with interactions with other genes or proteins that have pathogenic outcomes. Studies exploring the role of LRRK2 in the immune system were excluded, as this review focuses on LRRK2 in the context of PD.

**Figure 2:**
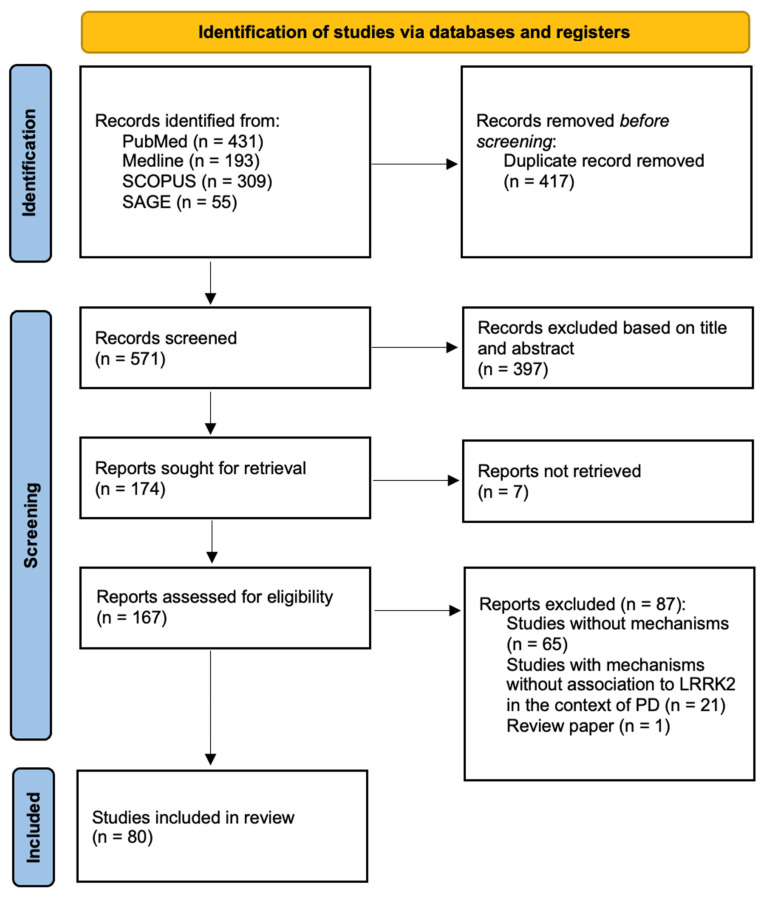
Flow chart of the literature search according to PRISMA guidelines [22].

The authors excluded articles based on the following criteria: review articles, abstracts, editorials/letters, conference proceedings, case reports, comments, imaging/biomarker studies for diagnostic purposes, and studies unrelated to PD or without LRRK2 as the gene of interest. A third author resolved differences of opinion between the two authors involved in the study selection. The flow of the methodology described is illustrated in Figure 2.

## 3. Results

A total of 988 records were identified, 431 found via Pubmed, 193 via Medline, 309 via SCOPUS, and 55 from SAGE. After combining search results from all databases, 417 duplicates were removed. After screening the titles and abstracts, 174 articles were sought for retrieval. In total, the full texts of 167 articles were assessed for eligibility based on the inclusion and exclusion criteria. Finally, 80 papers of in vivo and in vitro studies were included in this review, as seen in Figure 2.

Many of the papers included here involved the convergence of several mechanisms, so the authors decided to classify them according to what was perceived as the focus of each reviewed study, unless the focus was unclear. As such, there were 9 papers looking at α-Syn neurotoxicity, 3 on distinct inflammatory pathways, 4 involving glial–neuron crosstalk, 14 on mitochondrial dysfunction, 8 on vesicle trafficking, 6 on autophagy and lysosomal dysfunction, 4 on kinase signalling pathways, 3 on nucleus-related dysfunction, 2 on tau neurotoxicity, 3 on protein synthesis, 3 on Rab-LRRK2 interactions, 5 that were multifactorial, and 16 exploring factors that control LRRK2 expression and its pathogenicity. The literature exploring different molecular pathways of PD are summarized below.

### 3.1. LRRK2 and Alpha-Synuclein Neurotoxicity 

The abnormal aggregation of α-Syn has been long implicated in the pathogenesis of Parkinson’s disease. Aberrant accumulation of α-Syn forms Lewy bodies in the substantia nigra pars compacta (SNpc) and Lewy neurites—this is considered a hallmark of PD. While sporadic PD is largely characterized by the presence of Lewy bodies in surviving SNpc dopaminergic neurons, the presence of Lewy body pathology is pleomorphic in LRRK2-PD cases [23]. The relationship between LRRK2 and α-Syn toxicity in in vivo and in vitro models of Parkinson’s disease are summarized in Table 2. These studies reveal several ways that LRRK2 could potentially facilitate α-Syn neurotoxicity.

Several studies have demonstrated the role of LRRK2 in reducing α-Syn clearance in glial cells mediated by the endo-lysosomal pathway. In microglia, LRRK2 negatively regulates endocytosis pathways, as knockout of LRRK2 improves early endosomal formation by upregulating coordination between Rab5 and dynamin, and KO of LRRK2 also improves α-Syn clearance [24]. In LRRK2 G2019S astrocytes, the decreased capacity to degrade α-Syn is linked to loss of function of AnxA2, though it is not known how LRRK2 influences the levels of AnxA2 [31].

LRRK2 mediates α-Syn toxicity in neurons as well. Cultured neurons expressing LRRK2 G2019S have an enhanced mobility and inclusion of α-Syn [25]. This effect can be reversed by LRRK2 kinase inhibition. In another study, LRRK2 G2019S induced changes in lysosomal morphology and acidification. These changes coincided with an accumulation of α-Syn and increased neuronal release of α-Syn [32]. The α-Syn pathology was reversible by LRRK2 kinase inhibition.

Other factors in combination with LRRK2 impact α-Syn metabolism. Neuronal activity could be an additional driving force to the hyperactivity of LRRK2 kinase [30]. Age-related mechanisms may contribute to α-Syn metabolism. G2019S expression in dopaminergic neurons leads to age-related senescence via the p53-p21 pathway, which accelerates α-Syn aggregate formation [29]. Downstream effects of LRRK2, such as the phosphorylation of Rab35, mediates neuron to neuron propagation of α-Syn [26].

Some studies demonstrated the effects on neuronal death while studying LRRK2 and α-Syn. In LRRK2 G2019S transgenic mice, dopaminergic neuronal loss and increased endogenous phosphorylated α-Syn were observed in the substantia nigra [27]. The neuronal death and α-Syn pathology was not observed in transgenic mice with the kinase-dead form of LRRK2 G2019S, indicating the effects are kinase-dependent. In contrast, in a non-transgenic mouse model designed to exhibit α-Syn pathology, kinase inhibition by potent LRRK2 inhibitor MLi-2 was unable to protect neurons from death and α-Syn accumulation [28].

While the pathogenesis of α-Syn inclusions and subsequent Lewy body formation have not been fully understood, these studies suggest that LRRK2 plays a role in exacerbating the α-Syn neurotoxicity.

### 3.2. LRRK2-Mediated Neuroinflammation

Inflammation is a hallmark in neurodegenerative diseases and is implicated in Parkinson’s disease. LRRK2 may play a regulatory role in several inflammatory pathways, as summarized in Table 3.

Under normal physiological conditions, LRRK2 directly phosphorylates RCAN1 and increases its binding affinity to Tollip, a toll-like receptor. RCAN1 phosphorylation leads to a positive modulation of IL-1β inflammatory signalling through a calcineurin-independent pathway, and this activity is exacerbated by LRRK2 variants G2019S and R1441C [33]. In addition, LRRK2 G2019S increases the protein levels of the Receptor of Advanced Glycation End Products (RAGE) that enhances the interaction between advanced glycation end products (AGE) and RAGE. This contributes to neuronal loss through oxidative stress as well as inflammation [34]. R1441G and G2019S mutations increase the susceptibility of long-term lipopolysaccharide (LPS)-induced neuronal loss. Exposure to LPS increases neuroinflammation that is initiated by peripheral circulating inflammatory mediators, as opposed to direct initiation by T-cells or resident glial cells [35]. These studies provide evidence of inflammation-related pathways modulated by the wild-type LRRK2 and pathogenic LRRK2 mutations.

### 3.3. LRRK2-Linked Neurotoxicity Associated with Glial Cells

Glia contribute to the LRRK2-mediated αSYN neurotoxicity discussed in Section 2.1. The other ways glial cells can compromise neuronal survival are shown in Table 4.

Pathogenic astrocytes expressing G2019S can deplete healthy iPSC-derived ventral midbrain dopaminergic neurons when co-cultured together. Chaperone-mediated autophagy (CMA) and macroautophagy were compromised in mutant astrocytes [36]. Treatment with a CMA activator was able to restore functional proteostasis of α-Syn, however, it only led to partial recovery of neurodegeneration.

Kinase activity of LRRK2, elevated by G2019S, can regulate inflammatory signalling pathways in microglia. In microglia with the LRRK2 G2019S mutation, protein kinase A (PKA) activity was downregulated through a decrease in cAMP levels caused by increased phosphodiesterase 4 (PDE4) activity. PKA activity causes the accumulation of the inhibitory subunit of NF-κB p50. Therefore, this downregulation of PKA reduces NF-κB inhibitory signalling. As a result, in response to treatment with pre-formed fibrils of α-Syn, LRRK2 G2019S microglia had increased levels of pro-inflammatory IL-1β compared to wild-type microglia [38].

Transcriptome analyses of LRRK2 knockout microglia have suggested that LRRK2 plays a role in redox signalling in microglia. In response to pre-formed fibrils of αSYN, there was a decrease in the induction of the antioxidant Superoxide Dismutase 2 (SOD2) with LRRK2 absent [39].

Meanwhile, astrocytes derived from PD patients with the LRRK2 G2019S and Beta-glucocerebrosidase 1 (GBA1) N370S mutations reflect several hallmarks of PD by displaying an altered phenotype of increased Ca^2+^ levels and increased reactivity upon inflammatory stimulation, mitochondrial DNA maintenance defects, metabolic changes, and altered polyamine metabolism [37].

### 3.4. LRRK2-Mediated Mitochondrial Dysfunction and Endoplasmic Reticulum Stress

Mitochondria are chief organelles in maintaining the health of neurons, especially to meet neuronal energy demand. Thus, deviations in the activity, health, and life cycles of the mitochondria have detrimental contributions in neurodegenerative diseases such as PD. Furthermore, endoplasmic reticulum (ER) stress could also affect the mitochondria due to the physical and functional interactions between the mitochondria and the ER. The studies exploring mitochondrial dysfunction and ER stress in the context of LRRK2 are summarized in Table 5.

In neurons expressing LRRK2 G2019S, mtDNA damage was specific to dopaminergic neurons in the midbrain compared to cortical neurons. The damage induced was reversible upon kinase inhibition [40]. Similar mtDNA dysfunction including accumulation of 7S DNA, low mitochondrial DNA replication, high heavy strand transcription, and low mitochondrial DNA release was also observed in fibroblasts of idiopathic and LRRK2 G2019S PD patients [41]. However, not all defects in mitochondria are dependent on the kinase activity of LRRK2. Kinase inhibition was unable to recover changes such as the increase in mitochondrial velocity and motility and the decrease in mitochondrial respiration rate corresponding to decreased sirtuin deacetylase activity and decreased NAD^+^ levels [42].

Another method through which LRRK2 can cause mitochondrial dysfunction is through calcium dyshomeostasis. It has been demonstrated that kinase mutants of LRRK2 promote mitochondrial injury by increasing Ca^2+^ influx. The increased influx is caused by an enhanced expression of MCU and MICU1, which make up part of the mitochondrial calcium uniporter complex. The enhanced expression is concurrent with the activation of the ERK1/2 (MAPK3/1) pathway [43].

LRRK2 also has a role in delaying efflux of Ca^2+^. The delay of efflux, however, was not only specific to mutants of LRRK2 G2019S and Y1699C, knocking out LRRK2 as well as inhibiting LRRK2 had the same effect. This decrease in extrusion of Ca^2+^ via the Na^+^/Ca2^+^/Li^+^ exchanger (NCLX), a calcium antiporter, resulted in increased cell death. Upregulating NCLX via a cAMP/PKA-dependent activation mediated calcium release and was able to rescue neurotoxicity and subsequent neuronal cell death [44].

This calcium dysregulation linked to LRRK2 is not exclusive to the mitochondria. In LRRK2 G2019S iPSC-derived dopaminergic neurons, the global translation of the 5′ untranslated region (UTR) of mRNA is more efficient. In particular, there is increased translation of voltage-gated Ca2^+^ channel (VGCC) subunits. This increase in protein synthesis is associated with an upregulation of calcium transporters, leading to an influx of Ca^2+^ which results in neuronal stress as well as increased mitochondrial burden that adds to neuronal stress [45].

Several studies described the relationship between mitochondrial dysfunction and ER stress. In α-Syn-treated transgenic mouse astrocytes, LRRK2 G2019S displayed the capacity to inactivate sarco/endoplasmic reticulum Ca^2+^-ATPase (SERCA) by direct association. The inactivation causes a depletion of Ca^2+^ levels in the ER. The depletion causes a Ca^2+^ flux into the mitochondria as ER–mitochondria contacts are induced [46]. The maintenance of the ER–mitochondria contact sites which allow the transfer of calcium is regulated by LRRK2. These contacts are maintained with wild-type LRRK2 but degraded in the presence of the G2019S mutation or absence of LRRK2. LRRK2 regulates the phosphorylation of the mitochondrial E3 ubiquitin ligases via ER-localized protein kinase R-like endoplasmic reticulum kinase (PERK), thereby determining ER–mitochondrial tethering [47].

LRRK2 not only influences the acceleration of ER stress by ER–mitochondria contacts but also the regulation of ER stress response genes. In astrocytes, LRRK2 G2019S increases ER stress through the SHP (receptor)-PIAS1 (protein)-XBP1 (transcription factor) pathway. G2019S mutation increases the expression of SHP, stabilizing the PIAS1 which causes a decrease in transcriptional activity of XBP1. This causes cell death, as the transcriptional activity of XBP1 is required to induce genes that reduce ER stress by ER-associated protein degradation [48].

Aside from causing dysfunction of mitochondria, LRRK2 can also impede mitophagy. G2019S expression prevents the necessary removal of Miro to compromise mitophagy [49]. In addition, G2019S decreases PINK1-Parkin-dependent mitophagy through the mediation of Drp1 [50]. Similarly, with R1441G, due to the defects in phosphorylation of the Drp1-dependent pathway, mitophagy was impaired upstream from the MAPK/ERK pathway. However, kinase inhibition did not reverse the reduced clearance of damaged mitochondria [51]. Epigenetic factors such as histone acetylation can affect mitophagy. Fibroblasts from PD patients with G2019S mutation displayed increased mitophagy due to the activation of class III HDACs. Fibroblasts from patients with idiopathic PD, however, exhibited a downregulation of mitophagy due to a decrease in class III HDACs [52].

Mitochondrial biogenesis is also a process that is regulated by LRRK2. An ageing yeast study found that the expression of the ROC, COR, and kinase domain of LRRK2 inhibits mitochondrial biogenesis, resulting in a decrease in mitochondrial mass [53].

### 3.5. LRRK2 and Vesicle Trafficking Events

The ways that LRRK2 and its variants can alter or disrupt vesicle trafficking events are shown in Table 6.

LRRK2 alters microtubule-mediated vesicular transport processes through its GTP binding activities rather than its kinase activities. Pathogenic mutants of LRRK2 and kinase-inhibited LRRK2 cause an increased colocalization of LRRK2 with microtubules which are said to cause dysfunction in microtubule-mediated vesicular transport events [54].

LRRK2 also can alter synaptic vesicle (SV) trafficking. The expression of the R1441C variant in Drosophila induced PD-like phenotypes and was associated with an increase in phosphorylation of synaptojanin 1 (SYNJ1) [55]. In mouse dopaminergic neurons expressing G2019S, there is also an increased phosphorylation of SYNJ1, which disrupts the interaction between SYNJ1 and endophilin, a requirement for SV endocytosis. SYNJ1 loss of function has been associated with early onset PD [56]. In two patient-derived dopaminergic neuron lines expressing R1441C and R1441G, the dysregulation of SV endocytosis altered dopamine metabolism and resulted in toxic effects such as oxidized dopamine and its downstream effects, including increased αSYN and decreased lysosomal glucocerebrosidase activity [57]. The study found that LRRK2 kinase activity regulates the phosphorylation state of DNAJC6 (auxilin) in its clathrin-binding domain at Ser627. Auxilin is a gene implicated in synaptic vesicle endocytosis dysfunction in PD [57].

In addition to that, transcriptomics and proteomic analysis of iPSC-derived dopaminergic neurons revealed that endocytic pathways are dysregulated due to the G2019S mutation and globally led to impairment of clathrin-mediated endocytosis (CME) [58]. The dysregulation was associated with the downregulation of CME-related proteins and of Rab proteins Rab5b, Rab7a, and Rab10.

In dopaminergic neurons, the internalization of dopamine receptor-1 (DRD1) is impaired due to both G2019S and R1441C, resulting in a decreased rate of dopamine receptor-2 (DRD2) Golgi complex trafficking to the cell membrane [59]. Dopamine is not the only neurotransmitter in which LRRK2 affects its transport. G2019S altered SV trafficking dynamics and elevated neurotransmitter release of glutamate that is needed in excitatory synaptic transmission [60]. Synapsin I, a regulator of neurotransmitter release at synapses, was revealed to be a downstream target of LRRK2 phosphorylation, and when hyperphosphorylated, is less efficient at the release of neurotransmitters.

LRRK2 causes alterations in synaptic activity through the dysregulation of Ca-dependent activator protein for secretion 2 (CADPS2). In this study, LRRK2 and α-Syn were found to have contrasting effects. LRRK2 WT and LRRK2 G2019S increased CADPS2 expression, whereas α-Syn reduced CADPS2 on a transcriptional level. These two effects were still detrimental to neuronal health, and bringing CADPS2 back to physiological levels was neuroprotective [61].

### 3.6. LRRK2-Mediated Defects in Autophagy/Lysosome Pathways

LRRK2 has an important role in endo-lysosome biology and consequently the process of autophagy, as seen in Table 7. 

Autophagic defects are observed due to LRRK2 mutations. The R1441C mutation disrupts the normal binding of the LRRK2 protein to the a1 subunit of Vacuolar-type ATPase (vATPase), preventing the maturation of the autophagosome [62]. In addition to that, LRRK2 interrupts aggresomal formation for autophagic clearance. By inhibiting proteosome formation using MG-132, they effectively induced autophagy and found that in the presence of the G2019S mutation, aggresomal formation required for protein aggregate clearance was interrupted [63].

Downstream targets of LRRK2 also have a role in lysosomal dysfunction. Rab10 phosphorylation is associated with lysosomal overload stress. Particularly, it was the kinase activity of LRRK2 that was found to be a key regulator of the exocytic release of lysosomal content under lysosomal overload stress [64]. In addition to release of lysosomal content, increased LRRK2 kinase activity impairs endosomal maturation or trafficking, resulting in lysosomal dysfunction and deficits in proteostasis [65]. LRRK2 hyperactivation was also found to alter axonal AV transport by recruiting motor adaptor protein JIP4 to the AV membrane. This disrupted the formation of the autolysosome as the retrograde motility was interrupted. These defects were linked to the G2019S mutation as well as the overexpression of Rab29 [66].

The Golgi body, an organelle associated with the process of autophagy and lysosome formation, was also found to be impacted by pathogenic LRRK2. Specifically, a role of LRRK2 in the regulation of membrane fusion at the trans Golgi network (TGN) has been described. LRRK2 was found to interact with the Golgi-associated retrograde protein (GARP) complex to regulate endosome transport, and depletion of GARP was able to worsen neurodegeneration in a LRRK2 G2019S *C. elegans* model [67].

### 3.7. LRRK2 and Kinase Signalling Pathways

In vivo and in vitro models suggest that LRRK2 is an upstream regulator of mitogen-activated protein kinases (MAPK). MAPKs are critical for the regulation of cellular functions such as cell proliferation, survival, and differentiation, and they do so by signal transduction pathways. Studies relevant to LRRK2’s role in kinase signalling pathways are shown below in Table 8.

The apoptosis signal-regulating kinase 1 (ASK1)–p38 MAPK pathway was found to be a downstream target of LRRK2 through the phosphorylation of ASK1. Furthermore, by analysing LRRK2 G2019S iPSCs, it was found that the neuronal apoptosis induced by LRRK2 was prevented by ASK1 inhibition [68]. Another way in which LRRK2 is involved in MAPK-related pathways is through the activation of the Hep/d-JNK/d-Jun signalling pathway in a Drosophila model. Neurodegeneration of dopaminergic neurons occurred with the LRRK2 G2019S mutation due to the activation of Hep, which initiated the Hep/d-JNK/d-Jun signalling pathway [69]. 

While it is evident that there are ways in which LRRK2 regulates kinase signalling pathways, the reverse is also true. There are kinase signalling pathways that regulate the abundance of LRRK2. F-box and leucine-rich repeat domain-containing protein 18 (Fbxl18), a component of an Skp1-Cullin1-F-box ubiquitin ligase complex, selectively targets phosphorylated LRRK2 for degradation and this is enhanced by protein kinase C activation [70]. Fbx proteins have crucial roles in several important signalling pathways, including NF-κB, Wnt, and Hedgehog. With an overexpression of LRRK2, β-catenin is repressed, resulting in a downregulation of Wnt signalling. In contrast, the protective variant of LRRK2 R1398H strengthened Wnt signalling [71].

### 3.8. LRRK2 and Nucleus-Related Dysfunction

Mutations in LRRK2 such as G2019S can impact both the structure and function of the nucleus, as seen in Table 9. A new physiological role of LRRK2 is stabilization of the nuclear lamina and maintenance of nuclear envelope homeostasis by binding to Seven in absentia homolog (SIAH-1) and direct interaction with lamin A/C within the nucleus. G2019S expression and LRRK2 knockdown caused disruption in nuclear envelope integrity, and the effects were not ameliorated with kinase inhibition [72]. G2019S caused defects in the 14-3-3 protein-associated DAF-16 nuclear translocation, increasing neuron susceptibility to oxidative stress by the decreasing expression of stress resistance genes sod-3 and dod-3 in *C. elegans* [73]. Furthermore, the G2019S mutation caused hypertrophy of the nucleus, while the R1441C mutation altered the nuclear size of the dopaminoceptive striatal spiny projection neurons (SPNs) of mice. Inhibiting kinase activity of LRRK2 also led to similar defects, namely nuclear hypertrophy [74]. These results indicate that stable LRRK2 activity is required for the maintenance of nuclear integrity.

### 3.9. LRRK2 and Protein Synthesis

LRRK2 can impact other cellular processes such as protein synthesis, as shown in Table 10. Protein synthesis deficiency has been observed in the models of LRRK2-PD. G2019S causes deficits in translation, and this impairment of translation occurs in patients with sporadic and LRRK2 PD [75]. Aside from dysfunction in translation due to a suppression of protein synthesis, LRRK2 can also impact translation through the inhibition of microRNA (miRNA). Wild-type LRRK2 interacts with the Argonaute-2 protein (Ago2-containing RISC: RNA-induced silencing complex) and pathogenic mutants R1441G and G2019S and inhibits the activity of the miRNA Let-7a. This inhibition, however, can be blocked with co-expression of Tripartite motif-containing protein 32 (TRIM-32). On the other hand, TRIM-32-mediated neuronal differentiation and subsequent cell death was found to be reduced with co-expression of R1441G [76].

### 3.10. LRRK2 and Tau Neurotoxicity

Tau, a protein typically associated with Alzheimer’s disease, has an emerging role in the pathogenesis of LRRK2-PD. Studies describing the relationship of tau pathology and LRRK2 are summarized in Table 11.

HEK-293 cells expressing LRRK2 G2019S had an increase in intracellular Tau level, accumulation, and secretion of Tau independent of its kinase activity. LRRK2 contributes to tau pathology through proteosome impairment and not the autophagy–lysosome pathway [77]. Another study describes the contribution of LRRK2 to tau neurotoxicity through distinct means, specifically, the dysregulation of actin and mitochondrial dynamics [78]. LRRK2 expression promotes tau neurotoxicity by an overstabilization of F-actin and subsequent abnormal localization of Drp1, a mitochondrial fission protein. An increase in both wild-type LRRK2 and expression of the G2019S mutant promoted the oligomerization of LRRK2, hence resulting in a decrease in actin-severing abilities [78]. Interestingly, it has been described that the tau pathology in primary neurons is not affected by LRRK2 kinase activity [79]. 

### 3.11. Rab-Dependent Processes That Are Affected by LRRK2

Downstream effectors of LRRK2 are the class of GTPases called Rabs. Rabs are phosphorylated by LRRK2 and in the context of PD, this phosphorylation contributes to pathogenic outcomes, as shown in Table 12.

The phosphorylation of Rab10 can interfere with physiological processes such as ciliogenesis. In LRRK2 R1441G mouse embryonic fibroblasts (MEFs), ciliogenesis is disrupted by pRab10 and subsequent recruitment of RILPL1, a regulator of ciliar protein localization. This disruption prevented responses to the Shh signal in R1441G MEFs and G2019S iPSCs. Furthermore, cholinergic neurons in R1441C mice striatum had defects in ciliation. A decrease in ciliation in cholinergic neurons blocks Shh signals from dopaminergic neurons, an essential signal for the release of neuroprotective factors such as glial-cell-line-derived neurotrophic factor (GDNF) [80]. pRab10, elevated by LRRK2 R1441G or R1441C, also interferes with ciliogenesis by increasing levels of RILPL2, a binding partner of Myosin Va. The inhibition of ciliogenesis could be attributed to the increased retention of Myosin Va, an essential protein in initiating ciliogenesis [81]. Another Rab that is associated with PD is Rab35. In transgenic mice with R1441C and G2019S mutations, there was an overexpression of Rab35 in the substantia nigra [82]. In the same study, elevated protein levels of Rab35 promoted the aggregation and secretion of A53T α-Syn in SH-SY5Y cells after transfection. 

### 3.12. LRRK2 and Mechanisms That Co-Exist

There were some studies that had several focuses in the pathogenesis of PD, such as inflammation, α-Syn, and mitochondrial dysfunction, and thus were categorized in this section, as seen in Table 13.

In a model of synucleinopathy, LRRK2 caused inflammation by promoting a calcineurin-dependent pathway through the phosphorylation of Nuclear Factor of Activated T Cells 2 (NFATc2). Kinase inhibition was able to prevent neuroinflammation and neuronal loss linked to NFATc2, but the inhibition worsened α-Syn clearance [83].

In addition to neuroinflammation, the interaction between LRRK2 and α-Syn can lead to mitochondrial dysfunction. LRRK2 and α-Syn interaction has convergent effects of actin cytoskeleton, resulting in mitochondrial dysfunction. G2019S promotes the oligomerization of LRRK2, reducing actin-severing activity and promoting excess stabilization of F-actin. This overstabilization is associated with interactions with α-Syn to promote neuronal degeneration and downstream dysregulation of mitochondria, such as aberrant metabolism and mislocalization of Drp1 [84].

Some studies have demonstrated how G2019S facilitates neuroinflammatory responses in microglia by regulating mitochondrial dynamics. G2019S induced microglial activation and mitochondrial fission in microglia. This altered morphology of mitochondria was heightened by LPS stimulation and coincided with an increase in Drp1 and TNF-α release [85]. These effects have been shown to aggravate neuronal death and are dependent on the kinase activity of LRRK2 [86]. Negative impacts on the mitochondria are not specific to G2019S; R1441G causes mitochondrial damage associated with mitophagy, ER stress, and increase in lysosomal stress due to induction of macroautophagy [87]. 

### 3.13. Factors That Control the Expression of LRRK2 and Its Pathogenicity

There are several studies that explore upstream factors that can influence LRRK2 pathogenicity, as seen in Table 14.

Two studies using a neurotoxin, 1-methyl-4-phenyl-1,2,3,6-tetrahydropyridine (MPTP), as a PD model, have described the role of long non-coding RNA in controlling the expression and abundance of LRRK2. An increase in levels of Hox transcript antisense intergenic RNA (HOTAIR) coincided with an increased stability of LRRK2 mRNA, upregulating the expression of LRRK2 [88]. Another lncRNA, metastasis-associated lung adenocarcinoma transcript 1 (MALAT1), promoted neuronal apoptosis by the downregulation of miR-20505p, which led to an overexpression of LRRK2 [89].

Mutations in the LRRK2 protein itself influence PD pathogenic outcomes by regulation of LRRK2 activity. Mutations at the ROC domain, R1441G/C/H, decrease the GTPase activity of LRRK2 and cause an inability to form dimers, an essential step in switching “off” the GTPase domain of LRRK2 [90]. In contrast, N1437H, another mutation in the ROC domain decreases GTPase activity by stabilizing the homodimeric conformation of LRRK2 [91]. Serine 910 and 935 are well-studied residues reflective of in vivo LRRK2 kinase activity. In an in vivo mouse model, pathogenic mutations of LRRK2 resulted in a loss of phosphorylation sites at serine 910 and 935, required for the interaction of LRRK2 with members of the 14-3-3 family of scaffolding proteins. Though the lack of phosphorylation at s910 and s925 was not sufficient to induce neurodegeneration, this resulted in a decrease in dopamine-regulating proteins as well as a reduced number of astrocytes [92].

There are also LRRK2 mutations that are risk factors instead of penetrant variants for inherited PD, such as G2385R. This mutation destabilizes LRRK2, as it has a higher affinity for two proteins involved in proteasomal degradation, Hsc70 (chaperone) and carboxyl-terminus of Hsc70-interacting protein (E3 ubiquitin ligase CHIP). This favours intracellular degradation over proteasomal degradation, resulting in lower steady-state intracellular protein levels due to increased protein turnover of the mutant protein [93].

In addition to LRRK2 mutations, the conformation of LRRK2 may influence its pathogenicity. A recent study found that the C-terminal domain of LRRK2 with G2019S mutation was sufficient to trigger the neurodegeneration of dopaminergic neurons, though it did not increase phosphorylation of Rab10 [94]. Using labelling of LRRK2 dimers in situ, it was found that homodimeric mutant LRRK2 G2019S exhibited greater kinase activity compared to heterodimeric WT/G2019S and monomeric LRRK2 [95]. Furthermore, a study involving molecular dynamics stimulation showed that G2019S missense resulted in a decrease in flexibility and an increase in compactness of the kinase domain of LRRK2, resulting in an inclination for LRRK2 to remain in its active conformation instead [96].

While mutations have different impacts on LRRK2 activity, the type of death pathway activated was a common factor amongst mutations of LRRK2. Specifically, signalling through the FADD-dependent extrinsic pathway was required by all pathogenic mutants of LRRK2 to induce neuronal death [97]. Different parts of LRRK2 contribute to this induction of the extrinsic death pathway. Firstly, there is a physical association between the motif within the N-terminal armadillo repeat region (ARM) of the LRRK2 and C-terminal DD domain of FADD. This interaction is further stabilized by the FADD-interacting α-helix of the N-terminal of LRRK2. Secondly, this interaction enables caspase-8 to be activated. Thirdly, the activation of FADD/caspase-8 extrinsic death components occur. Finally, pro-apoptotic Bcl-2 proteins, Bid and Bax, are recruited to activate the intrinsic mitochondrial apoptotic machinery [98].

LRRK2 is also affected by other PD-associated genes. A relationship between PINK1, linked with early onset PD, was highlighted [99]. The PINK1 mutant G309D increased LRRK2 expression at the levels of protein and RNA. Conversely, wild-type PINK1 has an inhibitory effect on LRRK2 at the transcriptional level.

Natural variation may also have an impact on the disease phenotypes observed. A study conducted with an LRRK2 G2019S Drosophila model found modifier genes that influenced the phenotypic outcomes of locomotor dysfunction and neuron loss [100]. The genes pros, pbl, ct, and CG33506 modified age-related dopaminergic neuron loss and locomotor dysfunction associated with that. The first two, pros and pbl, enhance the locomotor dysfunction, while ct and CG33506 suppress the impairment.

Other molecules, such as Peroxiredoxin 2 (Prx2), a member of the Prx family of antioxidant enzymes, was found to be a novel interacting protein of LRRK2 that could bind to the COR domain. Prx2 was found to be an upstream inhibitor of LRRK2, as it could reduce pRab10 levels, a downstream effector of LRRK2 [101]. In the context of pathogenic conditions, there is a functional association of TXNIP (thioredoxin-interacting protein) and LRRK2 that inhibits the activity of thioredoxin-1 (redox proteins) and reduces the translocation of NRF2, which would prevent release of antioxidants such as Prx2. Furthermore, it was found that TXNIP significantly accelerates the accumulation of α-Syn in a G2019S model [102]. When considering interacting factors of α-Syn, synphilin-1 (SP1), a cytoplasmic protein, interacts with LRRK2 as well. However, instead of contributing to its pathogenicity, SP1 is antagonistic to the LRRK2 mutation. SP1 prevented neuronal death and reduced kinase activities of LRRK2, and this translated to increased survival and improved locomotor activity in G2019S Drosophila [103]. 

## 4. Discussion

In this systematic review, we screened 726 journal articles and selected 80 articles that contain information about the different aspects in the pathogenesis of LRRK2-associated PD, namely, α-Syn neurotoxicity, inflammation, glial–neuron crosstalk, mitochondrial dysfunction, vesicle trafficking, autophagy and lysosomal dysfunction, kinase signalling pathways, nucleus-related dysfunction, tau neurotoxicity, protein synthesis, Rab-LRRK2 interactions, multifactorial studies, and factors that control LRRK2 expression and its pathogenicity.

### 4.1. Main Mechanisms of LRRK2-Associated PD

The main finding in pre-clinical studies of LRRK2-PD is that elevated LRRK2 kinase activity or phosphorylation of targets is linked to various aspects of pathogenesis.

Firstly, LRRK2 causes α-Syn neurotoxicity by decreased clearance of α-Syn and increased propagation and aggregate formation of α-Syn in a kinase-dependent manner. In the transgenic models with G2019S, α-Syn neurotoxicity induction is reversible with LRRK2 kinase inhibition, but not reversible in non-transgenic models [28]. Intriguingly, in models of synucleinopathy, LRRK2 inhibition can restore neuronal loss and motor deficits but worsens α-Syn clearance [83]. The similarity between these two studies is that LRRK2 inhibition is ineffective in preventing α-Syn neurotoxicity in the absence of pathogenic LRRK2. This suggests that though LRRK2 hyperactivation is present in idiopathic PD, it is not a main contributor to α-Syn neurotoxicity, but rather other activities lead of LRRK2 lead to neurodegeneration. The differences in these findings could be attributed to different mouse models. The former utilized an α-Syn PFF PD mouse model, whereas the latter utilized human α-Syn overexpressing transgenic mice. While the studies in this review have attributed neurotoxicity to α-Syn inclusion, some studies suggest α-Syn aggregation is a protective mechanism [97]. Rather than being a causative factor, Lewy body pathology could be an accompaniment of neuronal death. Therefore, the reversal of neurotoxicity with LRRK2 kinase inhibition could be associated with other exacerbated activities of LRRK2.

LRRK2 kinase activity modulates calcineurin-independent and calcineurin-dependent pathways [33,83]. LRRK2 impacts both inflammatory pathways, as it can phosphorylate RCAN1, which is an inhibitor of calcineurin and activates NFATc2, a downstream substrate of calcineurin [104]. Other inflammatory pathways influenced by kinase activities of LRRK2 include upregulation of AGE-RAGE, which activates the NF-κB signalling pathway [34]. The NF-κB signalling pathway is indirectly upregulated in microglia by LRRK2, as LRRK2 decreases NF-κB inhibitory signalling by downregulating PKA activity [38].

In addition to contributing to inflammatory pathways, glia to neuron crosstalk induces neurotoxicity through other ways. In both astrocytes and microglia, both WT and pathogenic LRRK2 had a decreased ability to clear α-Syn [24,31]. This accumulation of α-Syn in astrocytes can be linked to how LRRK2-G2019S compromises CMA and macroautophagy [36]. LRRK2 G2019S astrocytes had a decreased ability in responding to oxidative stress through distinct means, SERCA inactivation and the SHP (receptor)-PIAS1 (protein)-XBP1 (transcription factor) pathway [46,48]. In LRRK2 KO microglia, there was a decrease in antioxidant response after treatment with pre-formed fibrils of αSYN [39]. These three results suggest that diversion of typical activities of LRRK2, i.e., the hyperactivation of kinase, will affect LRRK2 redox signalling. LRRK2 also facilitates microglial activation in a kinase-dependent manner [85]. A newer study found that LRRK2 G2019S impacts microglia response to inflammation by Rab8a function in iron uptake and transport [105].

Mitochondrial dysfunction and ER stress are the main mechanisms in PD. LRRK2 causes it through two main ways: calcium dyshomeostasis and defects in the mitochondria life cycle. Calcium dyshomeostasis in the mitochondria and ER was exacerbated by LRRK2 mutants G2019S, Y1699C, and R1441C and by the knockout of LRRK2 [43,44,45]. This shows that elevated LRRK2 activity will not exclusively impact calcium homeostasis, as a loss of physiological LRRK2 activity impacts calcium homeostasis as well. Mutations of LRRK2 such as G2019S and R1441G cause defects in the mitochondrial life cycle by the inhibition of mitophagy [49,50,51]. In two of the three studies, one involving G2019S and another with R1441G, kinase inhibition was unable to reverse the defects in mitophagy, suggesting that factors aside from kinase hyperactivity reduce mitophagy [49,51]. The R1441G study involved LRRK2 mutant mice, whereas the G2019S studies used patient fibroblasts. The difference in response to inhibition of kinase activity despite the utility of similar models could be because of differences in monitoring of mitophagy in human primary fibroblasts. The former study focused on measuring Miro1 intensity as opposed to mitophagy induction [49,50]. In general, the life cycle of the mitochondria is affected by LRRK2 because expression of the enzymatic core of LRRK2 alone (kinase and COR domain) leads to reduced mitochondrial biogenesis [53].

Many downstream substrates of LRRK2 are involved in vesicle trafficking. A delicate balance of activities of LRRK2 is needed in physiological conditions for vesicular transport, particularly in microtubule-mediated vesicular transport events [54]. The increase in phosphorylation of downstream factors of LRRK2 such as SJN1, auxilin, and Synapsin 1 is linked to dysfunction in synaptic vesicle trafficking [56,57,60]. Defects in autophagy and lysosomal processes are caused by LRRK2 kinase hyperactivity, disassociation of normal binding partners of LRRK2, altered recruitment of AV proteins, and phosphorylation of Rab10.

Downstream Rab GTPases of LRRK2, including Rab10, play a pertinent role in LRRK2-PD pathology. From the studies in this review, Rab5, Rab7, Rab10, Rab29, and Rab35 were linked to various aspects of PD pathogenesis in a kinase-dependent manner. Rab5 and Rab35 were linked to α-Syn neurotoxicity by the downregulation of endosomal formation [24,26,82]. The negative regulation of CME was due to Rab5, Rab7, and Rab10 [58]. Lysosomal stress and impaired autolysosome formation was linked to Rab10 and Rab29, respectively [64,66]. In addition, the interference of ciliogenesis was mediated by binding of RILPL1 to pRab10 [80,81]. A separate study found that pRab8a and pRab10 were associated with RILPL1 and pRab8a and pRab12 was linked to RILPL2-related cilia defects [106]. Other PD-related phenotypes linked to Rab GTPases include impaired mitophagy. The phosphorylation of Rab10 prevented a necessary accumulation of Rab10 at the mitochondria that was necessary to induce mitophagy [107]. Of the five Rab GTPases implicated in LRRK2-PD, Rab10 has a role in several mechanisms and was found to be relevant in other forms of familial PD, including PINK1 and GBA1 [107,108]. This suggests a convergence of pathways with Rab10 as a substrate. Furthermore, pRab10 elevation is detectable in clinical samples of both LRRK2-PD and iPD patients [109]. Studies have been conducted to investigate the mechanisms that influence the phosphorylation of Rab10 by LRRK2. PPM1H, a phosphatase, was found to be a negative regulator of Rab10 phosphorylation [110]. The differential distribution of PPM1H in tissues could be an explanation for the variation of pRab10 in clinical samples [109].

Lastly, a downstream target of LRRK2 is tau. Previous studies have shown the in-vitro hyperphosphorylation of tau by LRRK2 [111,112,113]. Expression of LRRK2 G2019S in *Drosophila* promoted the phosphorylation of tau by the recruitment of glycogen synthase kinase 3β (GSK3β) [114]. The accumulation of insoluble and phosphorylated tau was observed in a transgenic LRRK2 mouse model of tauopathy [113]. Studies in this review reveal contrasting findings to previous knowledge of LRRK2 and tau. Two studies, despite different cell types, show that tau pathology observed in LRRK2-PD is kinase-independent [77,79]. Rather than the LRRK2 kinase activity contributing to tau neurotoxicity, it is attributed to proteosomal impairment and actin polymerization [77,78]. Two separate in vivo studies found that the oligomerization of LRRK2 promoted by its mutant forms resulted in overstabilization of F-actin, which has consequences for the accumulation and spread of proteins such as tau and α-Syn [78,84]. Overall, despite a lack of consensus that the kinase activities of LRRK2 directly impact tau pathology, there is evidence that LRRK2 promotes the spread of tau pathology.

### 4.2. LRRK2 Role in iPD and Familial PD

Studies reported in this review show that in the presence or absence of pathogenic variants, LRRK2 can contribute to the pathogenesis of PD. Comparisons were drawn from SH-SY5Y cells transfected with LRRK2 mutants, fibroblasts of LRRK2-PD patients, and post mortem brains of sporadic PD patients. Elevated protein levels associated with increased susceptibility to mitochondrial calcium dysregulation were observed for all groups, suggesting a shared mechanism between LRRK2-PD and sporadic PD [43]. Furthermore, kinase inhibition was able to reduce translational impairment in fibroblasts derived from both LRRK2 familial and sporadic PD patients [75]. It was also demonstrated that in patients with idiopathic PD, there was enhanced kinase activity of WT LRRK2 which was associated with abnormalities in mitochondria and lysosomal function [115]. Therefore, targeting LRRK2 activities may have broad therapeutic utility in idiopathic PD, not only in those who carry a LRRK2 mutation.

### 4.3. Potential Biomarkers in LRRK2-PD

The most significant biomarker in the detection of PD would be the increased kinase activity of LRRK2. LRRK2 kinase activity can be quantified by its autophosphorylation at serine1292 or the levels of downstream pRab10. In a Norwegian cohort, elevated levels of s1292 were identified in male patients that were carriers of G2019S [116]. These elevated pS1292 levels were found in brain and urinary exosomes. Future studies are needed to investigate pS1292 levels in larger, more diverse cohorts and to explore other LRRK2-PD mutations.

In this review, some studies highlight the possibility of novel biomarkers in LRRK2-associated PD. In vitro and in vivo studies have identified the overexpression of Rab35 and linked it to α-Syn pathology [26,82]. In addition to that, elevated mtDNA damage was found in iPD and LRRK2-PD patient fibroblasts [41]. Impairment of translation was also observed in fibroblasts of iPD and LRRK2-G2019S patients [75]. Further studies with human cohorts are needed to assess if these biomarkers are detectable in patient serum.

### 4.4. Therapeutic Strategies for LRRK2-Associated PD

**Figure 3:**
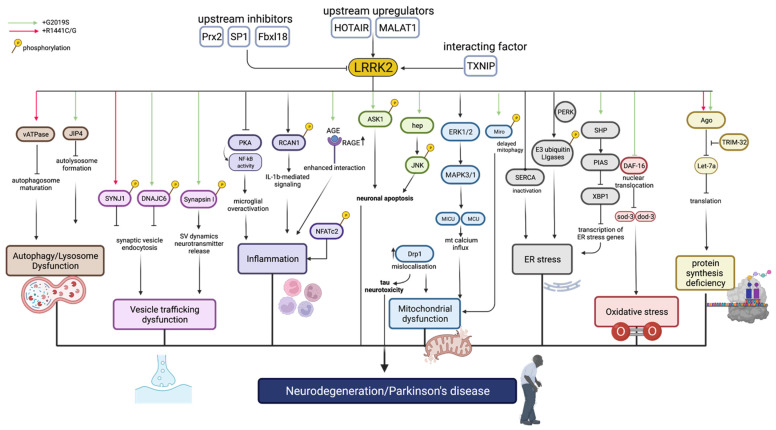
Targets of LRRK2 and associated mechanisms. Upstream and interacting factors of LRRK2 include Prx2, SP1, Fbxl18, HOTAIR, MALAT1, and TXNIP. Downstream factors related to autophagy and lysosomal dysfunction, vesicle trafficking, inflammation, mitochondrial dysfunction, ER stress, oxidative stress, and protein synthesis deficiency are shown. Green arrows represent targets associated with G2019S mutation, whereas red arrows are associated with R1441C/G mutations. Created with BioRender.com (accessed on 25 July 2022).

Figure 3 summarizes the list of upstream and downstream targets related to LRRK2 and associated mechanisms.

#### 4.4.1. Direct Inhibition of LRRK2

Table 15 states the type of interaction between LRRK2 and targets and identifies potential interventions for each target of LRRK2. There are various factors implicated in PD that are targets of LRRK2 phosphorylation. Therefore, direct inhibition of LRRK2 kinase activity is at the forefront of PD therapeutics, as it prevents the cascade of events leading to neuronal death. As of 2021, LRRK2 kinase inhibitors—DNL151 drug candidate of Denali Therapeutics—have undergone phase I of clinical trials [117].

Direct inhibition of LRRK2 kinase activity has a potential to impact other forms of familial PD. In this review, LRRK2 G2019S and GBA1-N370S-derived patient astrocytes shared hallmarks of PD [37]. Pre-clinical studies have shown that LRRK2 kinase inhibition can restore defects in GBA1 D490V mutant astrocytes [118]. The same group found that while defects caused by loss of GBA1 function can be attenuated by LRRK2 inhibition, the activity of the enzyme coded by GBA1, β-Glucocerebrosidase (GCase), is unaffected [119]. However, in that study, instead of mutant GBA1, GBA1-heterozygous-null iPSC-derived neurons were used, whereas in DA neurons derived from LRRK2-PD patients with G2019S or R1441C mutations, a reduction in GCase activity was observed in a manner mediated by Rab10 [120]. In contrast to the previous study, GCase activity in DA neurons with LRRK2 or GBA1 mutations can be increased with LRRK2 kinase inhibition. A study in 2022 corroborated these findings to some extent. In G2019S iPSC-derived neurons, GCase protein level was reduced, but in patient-derived fibroblasts and peripheral blood mononuclear cells, a positive correlation between LRRK2 kinase and GCase activity was found [108]. These studies indicate that LRRK2 kinase plays a role in regulating GCase activity, though there are differences specific to cell type. As these studies collectively suggest an interplay between these two PD-related genes, a case for GCase activation in LRRK2-PD can be also made.

While most of the studies in this review focus on the role of LRRK2 G2019S in PD, other variants of LRRK2 that are not directly linked to the kinase function of LRRK2 play a role in the pathogenesis of PD. Like G2019S, R1441G in the GTPase domain can cause mitochondrial defects through Drp1 [51]. In Figure 3, protein synthesis deficiency, vesicle trafficking dysfunction, and autolysosomal dysfunction are linked to R1441C/G mutations. Hence, inhibitors of the GTP binding activities of LRRK2 have been proposed as a different approach. However, at present only pre-clinical work justifies its therapeutic potential. In SH-SY5Y cells expressing LRRK2 R1441C, a GTPase domain mutation, impairments were seen in the neurite transport of mitochondria and lysosomes. Treatment with GTP-binding inhibitors were able to prevent these defects [121]. GTP-binding inhibitors could also reduce kinase activity. LRRK2 GTP-binding inhibitors 68 and Fx2149 promoted LRRK2 ubiquitination, increased ubiquitinated aggregation, and contributed to an aggresomal response. This could be linked to improved clearance of protein aggregates [122].

An alternative to reducing LRRK2 activity would be to reduce total levels of LRRK2 protein. Pre-clinical studies have shown that LRRK2 antisense oligonucleotides (ASO) can ameliorate α-Syn pathology, reduce elevated levels of ER Ca^2+^ and defects in mitophagy, and reduce locomotor deficits [123,124,125]. Furthermore, treatment of LRRK2 ASO in transgenic mice expressing LRRK2 G2019S or WT LRRK2 was able to decrease phosphorylation of Rab10 and correct autophagic defects caused by LRRK2 [125]. Currently, there is an ongoing phase I clinical trial for BIIB09, a LRRK2 ASO [126].

Studies exploring the physiological role of LRRK2 have described a role in immune response—T-cell function as well as the innate immune response [127,128,129]. LRRK2 kinase activity as a negative regulator of macroautophagy has been previously described [130]. Therefore, inhibition of LRRK2 activities or total reduction in protein could dysregulate immune or vesicle trafficking functions. Therefore, apart from direct inhibition of LRRK2 activity and protein level, potential pharmacological interventions involving inhibition or upregulation of LRRK2 targets could be considered.

#### 4.4.2. Indirect Inhibition of LRRK2

Inhibition of signalling pathways linked to neurodegeneration could be considered as a potential therapeutic intervention. As seen in Figure 3, there are signalling pathways activated or upregulated by LRRK2 linked to neurodegeneration. ASK1, an upstream regulator of P38 and JNK, when directly phosphorylated by LRRK2, results in neuronal apoptosis [68]. Therefore, ASK1 inhibitors which are currently undergoing clinical trials, some in phase III, could be used in the context of PD or broadly in neurodegenerative diseases [131]. Another inhibitor that is proposed are JNK inhibitors. These inhibitors, mainly in phase I trials, are being developed as anti-tumorigenic agents [132]. JNK inhibitors may be used in combination with other PD drugs. A study found that pathogenic LRRK2 results in JNK activation which led to motor impairment and neuronal death in a *Drosophila* model [69]. JNK inhibition alone only partially attenuated neurotoxicity [69]. Hence, combination therapy of LRRK2 inhibitors and JNK inhibitors would be more effective. Another signalling pathway that can be targeted is the AGE-RAGE intracellular signalling. G2019 enhances the interaction between AGE and RAGE, leading to inflammation and oxidative stress [34]. In a pre-clinical rat study, an anti-RAGE antibody was found to be able to block systemic inflammatory responses [133]. However, as it was unable to cross the blood–brain barrier, it may have limited applicability in the context of PD.

Upstream to LRRK2, as seen in Figure 3, there are genes causing its aberrant expression. Some in vitro studies have shown that the knockdown of these genes can attenuate neurotoxicity in LRRK2-associated PD [88,89]. Two long non-coding RNA, HOTAIR and MALAT1, increase the expression of LRRK2, thereby increasing its activity as well. Since gene knockdown is not feasible in humans, alternatives need to be explored. Both MALAT1 and HOTAIR have been proposed as therapeutic targets in cancer. Anti-lncRNAs were able to suppress HOTAIR activity in targeting solid tumours in vivo [134]. To suppress MALAT1 activity, siRNAs were able to elicit a degradation of MALAT1 [135]. A similar approach could be used to inhibit these lncRNA in PD models to assess its applicability. TXNIP is synergistic with LRRK2 and causes ER stress by preventing the release of antioxidants and is linked to α-Syn pathology. In human 3D midbrain organoids, inhibition of TXNIP was found to reduce LRRK2-induced phenotypes [102].

Another strategy is targeting downstream mechanisms such as mitochondrial dysfunctions (Figure 3). Firstly, pre-clinical work has shown that the reduction in Miro levels can attenuate neurotoxicity, as that allows damaged mitochondria to be degraded [136]. Secondly, the suppression of PERK can prevent degradation of ER–mitochondria contacts, which is necessary for mitochondrial health. In a mouse model for frontotemporal dementia, PERK inhibition was able to prevent further neuronal loss and lower levels of phosphorylated tau [137]. Thirdly, SERCA is a potential target as well. LRRK2 deactivates SERCA by direct association, which leads to ER stress and mitochondrial dysfunction [46]. As kinase inhibition of LRRK2 does not activate SERCA, another approach must be conducted, such as the pharmacological activation of SERCA through an allosteric activator. Current pre-clinical work utilizes SERCA activators for diseases such as type-2 diabetes and Duchenne Muscular Dystrophy [138,139]. Lastly, Drp1 inhibition could be a strategy as well to prevent neuronal death linked to mitochondrial dysfunction. A pre-clinical study found that Drp1 inhibition protected against neurotoxicity [140].

Antisense oligonucleotides (ASOs) are a potential strategy to reduce the total protein levels of LRRK2 and other interacting proteins. ASOs for α-Syn are being studied in pre-clinical models and have been found to prevent neurodegeneration associated with LRRK2 [141]. Another protein target implicated in LRRK2-PD, tau, which is abundant in carries of LRRK2 mutations, could also be addressed with a similar approach [23]. The use of tau ASOs could be necessary to address tau pathology in LRRK2-PD, as some studies have shown that it is LRRK2 kinase-independent [77,79].

While many of these targets are synergistic with LRRK2, there are some that are antagonistic targets (Figure 3). In upregulating the activity or levels of these targets, it may reverse or reduce the damage caused by overactivated LRRK2. There were two targets found to be upstream of LRRK2: Prx2 and Fbxl18. Prx2 was found to be an upstream inhibitor of LRRK2, as it could reduce pRab10 levels, a downstream effector of LRRK2 [101]. It is effectively an inhibitor of the kinase activities of LRRK2. Fbxl18 is able to target phosphorylated LRRK2 for degradation, which may be beneficial as self-phosphorylation of LRRK2 is also reflective of its kinase activities [70]. There were also factors that were not upstream of LRRK2 but could mediate the downstream effects of LRRK2. In pre-clinical studies, the upregulation of NCLX was able to help with Ca2^+^ influx caused by LRRK2 [44]. SP1 is a factor associated with α-Syn that has opposite effects on neuronal health compared to mutant LRRK2 [103]. Upregulating the activity of SP1 could be considered as a strategy in pre-clinical work. Lastly, the restoration of auxilin or DNAJC6 was able to prevent synaptic vesicle endocytosis dysfunction caused by LRRK2 [57]. Clathrin-mediated endocytosis (CME) is a key process mediated by auxilin; this could explain the functional antagonism between LRRK2 and auxilin [142].

**Figure 4:**
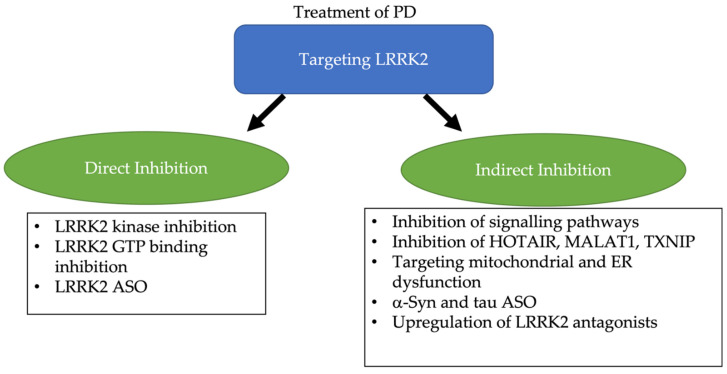
Summary of LRRK2 targeting strategies including direct inhibition, reducing LRRK2 activity and total protein levels, and indirect inhibition, inhibiting of downstream signalling pathways, inhibition of upregulation of LRRK2, targeting downstream effects, and upregulation of antagonists.

In summary, targeting LRRK2 for the treatment of PD can be divided into two categories, direct and indirect inhibition of LRRK2, with subcategories highlighted in Figure 4.

## 5. Conclusions

Studies over the past 6 years focusing on the mechanisms in LRRK2-associated PD have been identified. PD-causing variants of LRRK2 have been linked to similar and distinct mechanisms. While LRRK2 kinase hyperactivity is a main contributor to the dysfunctions observed, it is not a requirement for neurotoxicity. This review presents targets and gene interactions of LRRK2 that could be investigated further as an alternative to direct inhibition of LRRK2 activity. Several of the targets mentioned in this review are contributors in other diseases and therefore are worth considering in the context of PD.

## Figures and Tables

**Table 1:** Keywords used for the literature search.

Concept	Molecular Pathway(s)	LRRK2	Parkinson’s Disease
Synonyms	Mechanism	Leucine-rich repeat kinase 2	Parkinson’s
	Biological pathway(s)	PARK8	PD
	Pathogenesis	Dardarin	Paralysis agitans
	Signalling pathway(s)		
	Metabolic networks		

**Table 2:** Studies explore the relationship between LRRK2 and alpha-synuclein neurotoxicity.

Ref.	Study Model	Variant	Role of LRRK2/Molecular Pathway
[24]	in vitro, in vivo	WT	Reduces clearance of α-Syn in microglia by decreasing coordination of Rab5 and dynamin 1, essential for early endosome production
[25]	in vitro, in vivo	G2019S	Enhanced α-Syn mobility and inclusion
[26]	in vitro, in vivo	G2019S	Phosphorylation of Rab35 and dyshomeostasis of α-Syn aggregate trafficking through the endosomal pathway
[27]	in vivo	G2019S	α-Syn pathology is LRRK2-kinase-dependent (transgenic)
[28]	in vivo	-	α-Syn pathology is LRRK2-kinase-independent (non-transgenic)
[29]	in vitro, in vivo	G2019S	Senescence via p53-p21 pathway could accelerate α-Syn aggregate formation
[30]	in vitro, in vivo	G2019S	Neuronal activity can facilitate transmission and spreading of α-Syn
[31]	in vitro	G2019S	Astrocytes had a decreased capacity to clear α-Syn via the endo-lysosomal pathway due to annexin A2 (AnxA2) loss of function
[32]	in vitro, in vivo	G2019S	Accumulation of α-Syn and increased neuronal release of α-Syn

**Table 3:** Studies exploring the role of LRRK2 in neuroinflammatory pathways.

Ref.	Study Model	Variant	Role of LRRK2/Molecular Pathway
[33]	in vitro	WT, G2019S, R1441C	IL-1b-mediated signalling through regulator of calcineurin 1 (RCAN1)
[34]	in vitro, in vivo	G2019S	AGE-RAGE pathway
[35]	in vivo	G2019S, R1441G	peripheral immune signalling (type 2 IFN signalling)

**Table 4:** Studies investigating the contribution of glial cells to neurotoxicity associated with LRRK2.

Ref.	Study Model	Variant	Role/Molecular Pathway
[36]	in vitro	G2019S	chaperone-mediated autophagy and macroautophagy compromised
[37]	in vitro	G2019S	astrocytes exhibiting PD phenotypes
[38]	in vitro	G2019S	LRRK2 G2019S favours an overactive state of microglia through PKA-mediated NF-κB inhibitory signalling
[39]	in vitro	WT	induction of oxidative stress signalling in microglia

**Table 5:** Studies exploring the relationship of LRRK2, mitochondrial dysfunction, and ER stress.

Ref.	Study Model	Variant	Role/Molecular Pathway
[40]	in vitro	G2019S	mtDNA damage driven by LRRK2 kinase activity
[41]	in vitro	G2019S	mtDNA damage in fibroblasts of iPD and LRRK2 PD patients
[42]	in vitro	G2019S	Decreased sirtuin deacetylase activity, decreased NAD^+^ levels
[43]	in vitro	WT, R1441C, G2019S	Transcriptional upregulation of mitochondrial calcium uniporter (MCU) and Mitochondrial Calcium Uptake 1 (MICU1) by activation of the ERK1/2 (MAPK3/1) pathway (calcium dyshomeostasis)
[44]	in vitro	G2019S, Y1699C	NCLX activity and Ca^2+^ overload (calcium dyshomeostasis) linked with pKA activation
[45]	in vitro	G2019S	Calcium dysregulation due to aberrant protein synthesis (translatome alterations)
[46]	in vitro	G2019S	Disrupted Ca^2+^ homeostasis in the ER by interacting with SERCA
[47]	in vitro	G2019S	LRRK2 regulates ubiquitin ligase activity via PERK under ER stress
[48]	in vitro, in vivo	G2019S	ER stress is accelerated through the SHP/PIAS1/XBP1 axis
[49]	in vitro, in vivo	G2019S	LRRK2 pathogenic mutations delay mitophagy through the retention of Mitochondrial Rho GTPase (Miro)
[50]	in vitro	G2019S	PINK1-Parkin-dependent mitophagy via dynamin-related protein 1 (Drp1)
[51]	in vitro, in vivo	R1441G	Phosphorylation of DNM1L-MAPK/ERK was impaired in under mitochondrial stress
[52]	in vitro	G2019S	Increased mitophagy due to the activation of class III HDACs
[53]	in vivo	-	LRRK2 causes cell death through the inhibition of mitochondrial biogenesis

**Table 6:** Impact of LRRK2 on vesicle trafficking events.

Ref.	Study Model	Variant	Role/Molecular Pathway
[54]	in vitro	N1437H, R1441C, Y1699C,	alters microtubule-mediated vesicular transport processes through its GTP binding activities
[55]	in vitro	R1441C	Enhanced phosphorylation of SV proteins such as synaptojanin (SYNJ1) in the brain
[56]	in vitro	G2019S	Disrupt SV endocytosis by phosphorylation of SYNJ1
[57]	in vitro	R1441C/G	LRRK2 kinase activity regulates the phosphorylation state of DNAJC6 (auxilin) its clathrin-binding domain at Ser627
[58]	in vitro, in vivo	G2019S	Downregulation of CME proteins clathrin, endophilin, and dynamin and dysregulation of Rab5b, Rab7a, and Rab10 (needed for early endosome formation)
[59]	in vitro	G2019S	Impaired DRD1 internalization→ decreasing the rate of DRD2 trafficking
[60]	in vitro	G2019S	Altered SV dynamics and neurotransmitter release by Synapsin I hyperphosphorylation
[61]	in vitro	WT, G2019S	Increased CADPS2 gene and protein expression

**Table 7:** LRRK2 contributions to dysfunction in autophagy/lysosome pathways.

Ref.	Study Model	Variant	Role/Molecular Pathway
[62]	in vitro	R1441C	prevents maturation of autophagosome by preventing binding of LRRK2 to vATPase
[63]	in vitro, in vivo	G2019S	interrupts aggresomal formation
[64]	in vitro	WT, G2019S, R1441C, Y1699C	phosphorylates Rab10 leading to lysosomal overload stress
[65]	in vitro, in vivo	-	LRRK2 kinase activity impairs endosomal maturation or trafficking
[66]	in vitro	G2019S	recruits JNK-interacting protein 4 (JIP4) to the AV membrane, altering axonal autophagic vesicles (AV) transport
[67]	in vitro, in vivo	G2019S	interacts with the GARP complex at the trans-Golgi network, stabilizes the binding of GARP to the t-SNARE Syntaxin-6

**Table 8:** LRRK2 and kinase signalling pathways.

Ref.	Study Model	Variant	Role/Molecular Pathway
[68]	in vitro	G2019S	ASK1–p38 MAPK pathway
[69]	in vivo	G2019S	Hep/d-JNK/d-Jun signalling cascade
[70]	in vitro	-	Fbxl18 regulates LRRK2 abundance
[71]	in vitro, in vivo	-	alteration in Wnt signalling by repression of β-catenin

**Table 9:** Nucleus dysfunction and LRRK2.

Ref.	Study Model	Variant	Role/Molecular Pathway
[72]	in vitro, in vivo	G2019S	disruption in nuclear envelope (nuclear lamina)
[73]	in vivo	G2019S	decrease in expression of oxidative stress resistance genes due to inhibition of 14-3-3 protein-associated DAF-16 nuclear translocation
[74]	in vitro	G2019S, R1441C	nuclear structure changes hastening ageing process of neurons

**Table 10:** Defects in protein synthesis/translation associated with LRRK2.

Ref.	Study Model	Variant	Role/Molecular Pathway
[75]	in vivo	G2019S	Protein synthesis deficiency
[76]	in vitro, in vivo	WT, G2019S, R1441G	WT LRRK2 interacts with Ago-2 Pathogenic LRRK2 inhibits activity of miRNA, Let-7a LRRK2 inhibits TRIM-32 mediated neuronal differentiation

**Table 11:** Tau neurotoxicity in the context of LRRK2 models.

Ref.	Study Model	Variant	Role/Molecular Pathway
[77]	in vivo	G2019S	Proteosome impairment with G2019S leading to tau pathology
[78]	in vitro	WT, G2019S	Decrease in actin-severing, abnormal localization of Drp1
[79]	in vitro	-	Tau pathology kinase independent

**Table 12:** LRRK2 and Rab interactions in pathogenic context.

Ref.	Study Model	Variant	Role/Molecular Pathway
[80]	in vitro, in vivo	G2019S, R1441G/C	pRab10 binds to Rab-interacting lysosomal protein-like 1 (RILPL1) to inhibit ciliation, blocks Sonic hedgehog (Shh) signalling
[81]	in vitro, in vivo	R1441G/C	pRab10 increases binding of Rab-interacting lysosomal protein-like 2 (RILPL2) to Myosin Va, interfering with ciliogenesis
[82]	in vitro, in vivo	G2019S	Rab35 overexpression linked to α-Syn pathology

**Table 13:** LRRK2 and studies involving multiple mechanisms.

Ref.	Study Model	Variant	Role/Molecular Pathway
[83]	in vitro, in vivo	-	NFATc2 activation, calcineurin-dependent pathways
[84]	in vitro, in vivo	G2019S	interaction with α-Syn to promote neuronal degeneration through convergent effects on the actin cytoskeleton and downstream dysregulation of mitochondrial dynamics and function
[85]	in vitro	G2019S	microglial mitochondrial alteration via Drp1
[86]	in vivo	G2019S	proinflammatory microglia role in neuroinflammation and mitochondrial dysfunction
[87]	in vitro	R1441G	exhibits mitochondrial dysfunction, ER stress, and lysosomal stress

**Table 14:** Factors that control LRRK2 expression and its pathogenicity.

Ref.	Study Model	Variant	Role/Molecular Pathway
[88]	in vitro, in vivo	-	HOTAIR upregulates LRRK2
[89]	in vitro, in vivo	-	MALAT1 upregulates LRRK2
[90]	n/a	R1441/G/C/H	decreased GTPase activity of LRRK2 by impairing conformation
[91]	n/a	N1437H	decreased GTP binding affinity by promoting stable homodimer conformation
[92]	in vivo	-	loss of phosphorylation sites at serine 910 and 935
[93]	in vitro	G2385R	destabilizes LRRK2
[94]	in vitro, in vivo	G2019S	C-terminal of LRRK2 sufficient to induce neurodegeneration independent of Rab10 phosphorylation
[95,96]	n/a	G2019S	homodimeric G2019S has higher pathogenicity than heterodimeric G2019S G2019S decreases flexibility and increases compactness of LRRK2
[97,98]	in vitro in vivo	-	signalling of FADD-dependent extrinsic pathway is a requirement for neuronal death in LRRK2 mutants
[99]	in vitro	-	PINK1 mutant ↑ LRRK2 expression both at the RNA and protein levels
[100]	in vivo	G2019S	LRRK2 interactions with different background genetic modifiers modify phenotypic outcomes
[101]	in vitro, in vivo	G2019S	Prx2 is an upstream inhibitor of LRRK2
[102]	in vitro	G2019S	TXNIP interaction with LRRK2 prevents release of antioxidants TXNIP (thioredoxin-interacting protein) mediates the LRRK2-G2019S pathological phenotypes, significantly accelerates the accumulation of α-synuclein; in addition, TXNIP inhibits the activity of thioredoxin-1 (redox proteins) and ↓ translocation of NRF2.
[103]	in vitro, in vivo	G2019S	SP1 neuroprotective against LRRK2 kinase hyperactivity

**Table 15:** Summary of interacting factors of LRRK2 and associated mechanisms.

Ref.	Name of Target	Type of Interaction	Proposed Intervention
[83]	NFATc2	LRRK2 selectively phosphorylating and inducing nuclear translocation	LRRK2 kinase inhibitor
[33]	RCAN1	Phosphorylation	LRRK2 kinase inhibitor
[34]	AGE-RAGE	LRRK2 enhances the interaction	Anti-RAGE antibody
[38]	PKA	Downregulates PKA activity through PDE4	LRRK2 kinase inhibitor
[43]	MCU MICU1	Enhances MCU and MICU1	NCLX upregulators
[46]	SERCA	Inactivation of SERCA by direct association	SERCA activators
[47]	E3 ubiquitin ligases	Phosphorylation via PERK	Suppression of PERK/LRRK2 kinase inhibitor
[50]	Drp1	Unclear but Drp1 increases and is mislocalized	Drp1 inhibitor
[49]	Miro	Retention	Promotion of Miro degradation
[48]	SHP	Increases expression, which causes XBP1 SUMOlyation	doxycycline
[57]	DNAJC6 (Auxillin)	Phosphorylation	LRRK2 kinase inhibitors
[60]	Synapsin I	Phosphorylation	LRRK2 kinase inhibitors
[62]	vATPase	Normal binding of LRRK2 protein to a1 subunit of vATPase prevented with R1441C	LRRK2 kinase inhibitors
[64,80]	Rab10	Phosphorylation leading to lysosome and cilia formation dysfunction	Prx2 or LRRK2 kinase inhibitors
[101]	Prx2	Binds to COR domain of LRRK2, able to inhibit LRKR2 activity	Prx2 upregulation
[66]	JIP4	Recruitment of JIP4 to AV membrane→disrupt autolysosome formation	LRRK2 kinase inhibitors
[68]	ASK1	Direct phosphorylation of ASK1	ASK1 inhibition
[69]	hep	JNK phosphorylation (activation)	JNK inhibition
[73]	DAF-16	Inhibition of nuclear translocation associated with 14-3-3 proteins	LRRK2 kinase inhibitor or 14-3-3 protein upregulation
[67]	Ago2	Direct interaction which inhibits activity of miRNA Let-7a	LRRK2 kinase inhibitor
[76]	TRIM-32	Inhibition (affects neuronal differentiation)	LRRK2 kinase inhibitor
[88]	Fbxl18	Targets phosphorylated LRRK2 for degradation and this is enhanced by protein kinase C activation	Upregulate Fbxl18
[102]	TXNIP	TXNIP is upstream of LRRK2, interacts to inhibit the activity of thioredoxin-1 (TRX-1) and lower translocation of NRF2	Downregulate TXNIP
[88]	HOTAIR	Stabilizes expression of LRRK2 mRNA	Knockdown of HOTAIR
[89]	MALAT1	Overexpression of LRRK2	Knockdown of MALAT1
[103]	SP1	Antagonistic to LRRK2	Upregulation of SP1

## Data Availability

Not applicable.
